# An Incidental Malignant Gastrointestinal Neuroectodermal Tumor of the Stomach: A Rare Case Report and a Literature Review

**DOI:** 10.7759/cureus.28042

**Published:** 2022-08-15

**Authors:** Bahaaeldin Youssef, Rawan M Mohamed, Parisa Vahhabaghai, Don Asberry

**Affiliations:** 1 Pathology, East Tennessee State University, Johnson City, USA; 2 Public Health, East Tennessee State University, Johnson City, USA

**Keywords:** gastrointestinal neuroectodermal tumor, gnet, gastric gnet, clear cell sarcoma of the gastrointestinal tract, ewsr gene, ewsr1-atf1, ewsr1-creb1

## Abstract

Gastrointestinal neuroectodermal tumors (GNETs) are malignant tumors frequently arising within the muscularis propria of the gastrointestinal tract and often extend into the submucosa and subserosa. The stomach is the second most common site of incidence of GNETs after the small intestine. The most important differential of GNET is the clear cell sarcoma-gastrointestinal (CCS-GI). Both share similar morphological as well as molecular features and show S100 positivity; however, the lack of melanocytic differentiation in GNET distinguishes it from CCS-GI. Both typically show rearrangements of the *EWSR1* gene, with t(12;22) (q13;q12) *EWSR1-ATF1* or t(2;22) (q34;q12) *EWSR1-CREB1* fusions. We present a case of a 71-year-old man with an incidentally discovered GNET in the gastric cardia and fundus.

## Introduction

A malignant gastrointestinal neuroectodermal tumor (GNET) is a rare and aggressive tumor that typically occurs in adults, most frequently occurring in the stomach and the small intestine. Other common sites are the esophagus, colon, and anal canal [1]. GNETs typically involve the muscularis propria of the gastrointestinal wall and often extend deeper to the submucosa and subserosa. GNET may present as a polypoid mass that causes luminal stenosis due to wall thickening. Histologically, the neoplastic cells show epithelioid or spindled morphologic patterns. The epithelioid cells are frequently arranged in nests or may possess sheet-like, papillary, or pseudoalveolar configurations. Nuclei exhibit significant pleomorphism with abundant eosinophilic or clear cytoplasm [2]. The spindle tumor cells are arranged in a fascicular pattern with abundant eosinophilic cytoplasm. Their histopathologic features suggest an origin from a gastrointestinal neuroectodermal precursor cell that fails to differentiate along the melanocytic lineage [3].

## Case presentation

**Figure 1:**
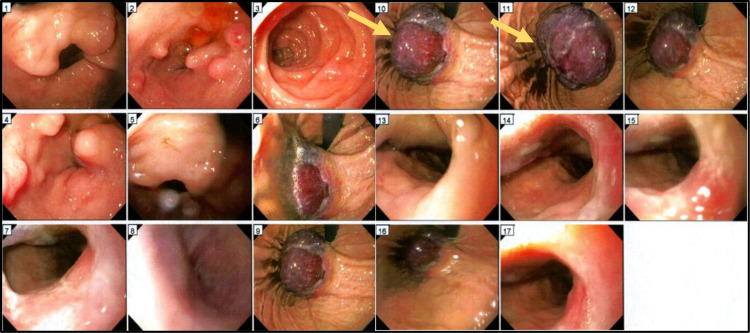
Esophagogastroduodenoscopy (EGD) A 7.8 cm mass (Arrow) was detected in the gastric cardia and fundus.

We report the case of a 71-year-old man with a past medical history of hypertension, hyperlipidemia, and benign prostatic hyperplasia who presented with complaints of dyspnea, fatigue, black stools, and a recent syncopal episode. Laboratory testing revealed anemia (HB 5.4 g/dl). A subsequent esophagogastroduodenoscopy was performed, and a submucosal gastric mass was detected (Figure 1). An abdominal CT scan involved a 7.8 cm mass along the gastric cardia and fundus.

**Figure 2:**
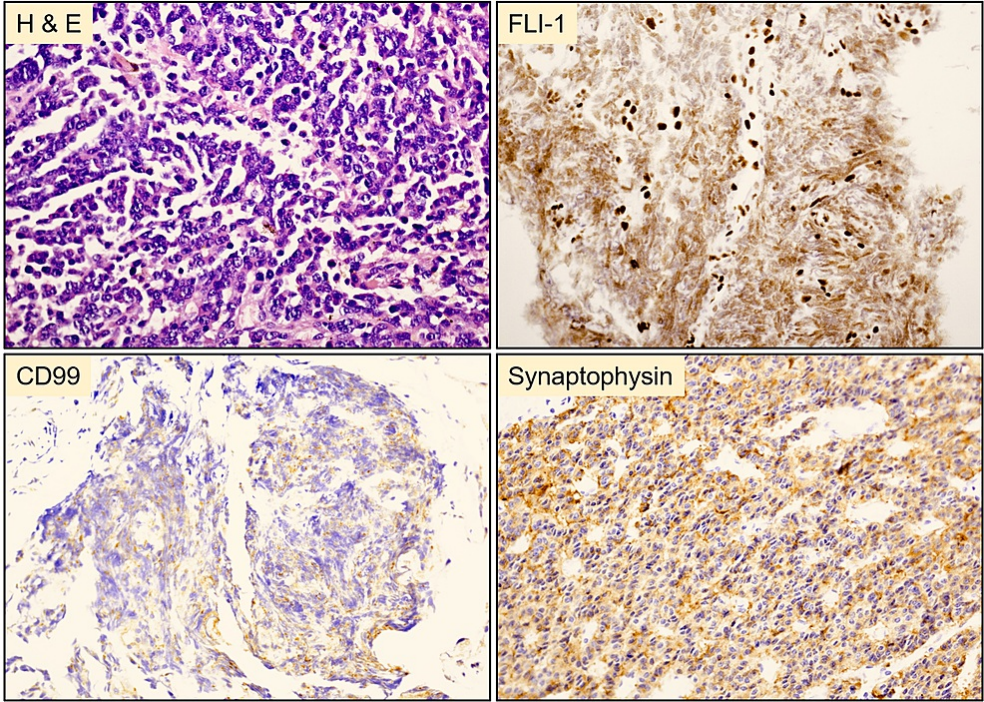
Histopathology of the resected tumor from the stomach Polygonal tumor cells with round to oval nuclei and modest eosinophilic cytoplasm arranged in a fasicular pattern. Tumor cells were positive for FLI-1, CD99 (weak), and synaptophysin.

**Figure 3:**
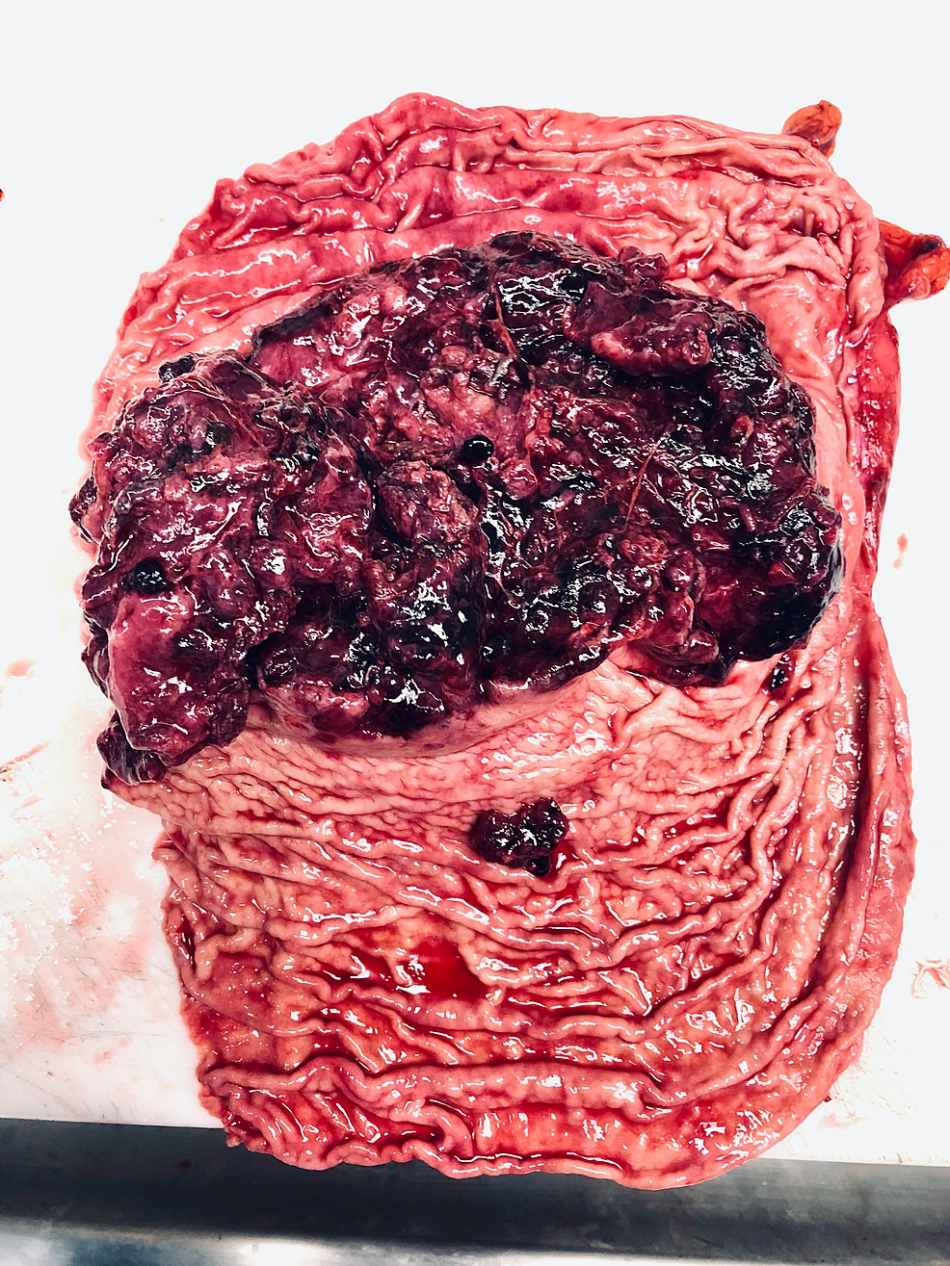
Gross photomicrograph of the mass in the stomach following gastrectomy The tumor (7.8 x 3.6 x 1.9 cm) was red and polypoid with multifocal surface ulceration.

The mass was biopsied for histopathologic examination. Microscopically, the neoplastic cells were arranged in a fascicular pattern with a round to oval nuclei and modest eosinophilic cytoplasm. Immunohistochemically, the tumor cells were positive for synaptophysin, Fli-1, and CD99 and negative for HMB45, MART-1, chromogranin, TTF-1, SMA, desmin, CD34, EMA, Keratin AE1/AE3, and CD45 (Figure 2). The histopathologic and molecular features were consistent with a malignant gastrointestinal neuroectodermal tumor. Fluorescence in situ hybridization (FISH) analysis revealed the splitting signal in 163 out of 200 nuclei indicating the presence of *EWSR1 *gene rearrangement, confirming the rendered diagnosis. The mass was surgically resected following the diagnosis, and clinical follow-up two months later confirmed metastatic deposits in the liver and spleen by computed tomography scan (CT) (Figure 3). The patient died one week after confirming the distant metastasis.

## Discussion

GNETs are very rare mesenchymal neoplasms of the gastrointestinal tract without a well-defined etiology. Common sites of involvement include the small bowel, stomach, or colon. GNET is thought to arise following a prolonged treatment course of a primary tumor (such as Hepatoblastoma, Ewing sarcoma, or a primary gastric adenocarcinoma) with the conventional treatment modalities (Surgery plus chemotherapy or radiotherapy). It hypothesized that chemotherapy might be a potential trigger for the onset of GNET [4]. GNET is an aggressive tumor that tends to occur in young to middle-aged adults without a specific gender predilection and shows a high likelihood of recurrence. Most tumors are metastatic to the regional lymph nodes or the liver by the time of presentation, with an average survival time of 18 months [5,6]. 

The clinical presentation varies from symptoms of anemia, weight loss, and abdominal pain to an abdominal mass that can cause intestinal obstruction and is usually detected by a CT scan [7,8]. Gross examination of the tumor usually shows an endophytic mass that gradually infiltrates the mucosa. Microscopically, the neoplastic cells exhibit a spindle-shaped or epithelioid morphology with eosinophilic or clear cytoplasm. Architectural patterns include nests or sheets of cells that can sometimes show an alveolar or a pseudopapillary pattern. They have an irregular nuclear membrane with abundant mitotic figures and frequent necrosis [9-11]. Immunohistochemically, GIST exhibits a strong and diffuse immunoreactivity for S100 protein, SOX10. CD56 and synaptophysin are generally positive. NB84, neuron specific enolase and neurofilament can be positive.

Different pathologic entities exist in the differential diagnosis of GNET. This poses a diagnostic challenge for distinguishing GNET. Gastrointestinal stromal tumors (GIST) and GNET share clinical and histomorphology features as both arise in the wall of the gastrointestinal tract and microscopically show spindle or epithelioid cells. Also, GIST is frequently positive for S100. It is important to exclude metastatic melanoma or clear cell sarcoma of the gastrointestinal tract (CCS-GI). Immunohistochemical staining would aid in the differential diagnosis since GISTs are negative for melanocytic markers such as HMB45, Melan A, and tyrosinase and are also negative for CD34, CD117, and DOG-1. Molecular characterization of the tumor nature will conclude the final diagnosis in the context of the clinical and histologic picture. GNET and GIST share some characteristics where both tumors arise in the gastrointestinal tract wall and microscopically show spindle and/or epithelioid cells. However, GIST does not typically show osteoclast-like multinucleated giant cells [12]. Malignant giant cell tumors and leiomyosarcoma are also included within the list of differentials that need to be excluded. Both entities arise in the bowel and have an osteoclast-type population; however, neither stains with S-100 [13,14]. GNET typically shows rearrangements of the *EWSR1 gene*, with t(12;22) (q13;q12) *EWSR1-ATF1* or t(2;22) (q34;q12) *EWSR1-CREB1* fusions with no specific impact on prognosis or clinical management [15,16]. GNETs tend to have a poor prognostic outcome. Surgical resection is the treatment of choice for GNET. Regional lymph node dissection, radiotherapy, and adjuvant chemotherapeutic regimens are the treatment options for metastatic disease. 

## Conclusions

GNETs pose a diagnostic challenge both clinically and microscopically, where the careful distinction of the histologic and immunohistochemical patterns is warranted. Pathologists show be aware of this clinical entity when considering the differential diagnosis of a gastric mass showing spindle or epitheliod morphology. 
